# Preparation of Highly Antibacterial MXene Nanofiltration Membranes and Investigation of Their Separation Performance

**DOI:** 10.3390/polym17111493

**Published:** 2025-05-27

**Authors:** Na Meng, Jinxin Liu, Jialing Mi, Xuan Chen, Rong Rong, Junjie Hang, Zihan Jiang

**Affiliations:** 1 Jiangsu Key Laboratory of Industrial Pollution Control and Resource Reuse, School of Environment Engineering, Xuzhou University of Technology, Xuzhou 221018, China; ljx2667495913@163.com (J.L.); 17751282269@163.com (J.M.); rongrong114717@163.com (R.R.); 13961965121@163.com (J.H.); w2791957440@163.com (Z.J.); 2 School of Technology-Saint Pertersburg Joint Engineering, Xuzhou University of Technology, Xuzhou 221018, China; chenxuan2645@163.com

**Keywords:** PES/SPES membrane, blend membrane, Ti_3_C_2_T_X_, antibacterial activity

## Abstract

In this study, polyethersulfone (PES)/sulfonated polyethersulfone (SPES) composite nanofiltration membranes doped with different contents of monolayer titanium carbide nanosheets (Ti_3_C_2_T_X_) were prepared by the nonsolvent induced phase inversion (NIPS) method. The effects of Ti_3_C_2_T_X_ on membrane structure, separation performance and antibacterial activity were investigated systematically. The results demonstrated that the viscosity of the casting solution increased significantly with the increasing content of Ti_3_C_2_T_X_. In addition, the pore size of the membrane surface first decreased and then increased; porosity and hydrophilicity were optimized synchronously; and the density of negative charges on the surface increased. The M2 membrane showed a rejection rate of more than 90% for Metanil yellow (MY) and methylene blue (MEB). The order of salt ion rejection rates was magnesium sulfate (MgSO_4_) > sodium sulfate (Na_2_SO_4_) > sodium chloride (NaCl), and water flux reached the peak (18.5 L/m^2^·h·bar). The antibacterial activity of the M2 membrane was significantly enhanced, and its antibacterial rate against *Bacillus subtilis* increased from 15% (M0) to 58%. This phenomenon was attributed to the synergistic mechanism of the Ti_3_C_2_T_X_ physical capture effect, reactive oxygen species (ROS) generation and sharp edge damage to bacterial cell membranes. This study provides theoretical support and a technical path for the development of MXene composite membranes with high separation efficiency and excellent antibacterial properties.

## 1. Introduction

As a high-performance polymer material [1], polyethersulfone (PES) exhibits excellent thermal stability due to the absence of -C-C- chains or biphenyl structures and the presence of only sulfone groups (-SO_2_-), ether linkages and aromatic rings in the PES molecular structure [2,3]. Additionally, it is widely used in a variety of fields because of its alkali and pressure resistance. However, its inherent hydrophobicity limits its application in membrane separation technologies.

Previous studies have improved the hydrophilicity and separation performance of PES membranes by incorporating hydrophilic materials. For instance, Athira et al. (2020) blended PES with sulfonated polyethersulfone (SPES) and cellulose acetate (CA) to enhance the hydrophilicity and biocompatibility of membranes [4]. Li et al. employed the phase inversion method to prepare PES/SPES membranes and investigated the effects of SPES molecular weight and blending ratio on membrane properties. Their results indicated that higher SPES molecular weight improved hydrophilicity and surface negative charge density [1]. Inspired by electric eels, Huang et al. fabricated PES/SPES membranes with abundant nanochannels for salinity gradient power generation and achieved promising results [2]. Almanassra et al. synthesized metal–organic framework (MOF) nanoparticles, incorporated them into acid-treated hybrid membranes and optimized physicochemical properties like hydrophilicity [5]. In 2024, Kadadou et al. modified PES membranes with polydopamine (PDA) and Ce@MOF and enhanced hydrophilicity, pore size and porosity [6]. Overall, blending hydrophilic materials into PES-based membranes improves their hydrophilicity and separation performance to a large extent.

MXene is a new two-dimensional material from the family of transition metal carbides, nitrides or carbonitrides. In terms of membrane separation, it has drawn widespread attention by virtue of its high conductivity, excellent mechanical properties, thermal stability, hydrophilicity, surface functionalizability and large specific surface area. Researchers have also discovered that MXene membranes exhibit remarkable antibacterial properties. For example, Kashif et al. deposited monolayer titanium carbide nanosheets (Ti_3_C_2_T_X_) onto polyvinylidene fluoride (PVDF) membranes via vacuum filtration and achieved an inhibition rate of over 99% against *E. coli* and *Bacillus subtilis* [7]. Similarly, mixed-matrix membranes were fabricated by Lu et al. using modified carbon nanotubes, MXene and PES, and demonstrated nearly 100% antibacterial activity against *E. coli* [8]. However, the antibacterial performance of the PES/SPES/Ti_3_C_2_T_X_ mixed-matrix membrane against *Bacillus subtilis* has not been tested so far.

In this study, nonsolvent induced phase inversion (NIPS) was used for preparing PES/SPES composite nanofiltration membranes by incorporating hydrophilic Ti_3_C_2_T_X_ into the casting solution. The influence of Ti_3_C_2_T_X_ on the microstructure and separation performance of composite membranes was systematically investigated. This work provides insights for advancing membrane technology in dye/salt separation applications.

## 2. Experiments

### 2.1. Materials

Ti_3_C_2_T_X_ was purchased from Jiangsu XFNANO Materials Tech Co., Ltd. (Nanjing, China). Sodium sulfate (Na_2_SO_4_), magnesium sulfate (MgSO_4_) and sodium chloride (NaCl) were provided by Sinopharm Chemical Reagent Co., Ltd. (Shanghai, China). N,N-Dimethylacetamide (DMAc), methylene blue (MEB), methyl blue (MB), methyl orange (MO), Metanil yellow (MY) and safranine T (ST) were bought from Shanghai Aladdin Biochemical Technology Co., Ltd. (Shanghai, China) PES was supplied by Shuangfu Plastic Trading Co., Ltd. (Foshan, China). SPES was synthesized in the laboratory. Deionized water was used throughout the experiments.

### 2.2. Preparation of Nanofiltration Membranes

The NIPS method was adopted to prepare PES/SPES composite membranes. The compositions of the casting solution are listed in Table 1. First, a magnetic stirrer was used to dissolve PES, SPES and Ti_3_C_2_T_X_ for 14 h and form a homogeneous casting solution. After degassing to eliminate bubbles, the solution was cast onto a clean glass plate using a casting knife to obtain a film with a thickness of 100 μm. The film was immersed in a coagulation bath (deionized water) at once for the removal of residual solvents. The formed membrane was subjected to 24 h storage in the coagulation bath to ensure the complete removal of solvents, then dried and stored for further use. The membranes were labeled as M0, M1, M2 and M3 based on Ti_3_C_2_T_X_ content.

### 2.3. Characterization of Nanofiltration Membranes

Scanning electron microscopy (SEM, Hitachi Regulus8100, Hitachi, Tokyo, Japan) was used to observe the surface and cross-sectional morphologies of membranes. A contact angle analyzer (Dataphysics OCA20, Filderstadt, Germany) was utilized for measuring the hydrophilicity of membranes, with at least two measurements per sample. Atomic force microscopy (AFM) was applied to analyze surface topography and roughness (Bruker Dimension ICON, Bruker, Munich, Germany). A solid-surface zeta potential analyzer was leveraged to determine the surface charge (zeta potential) of membranes (Anton Paar surpass3, Graz, Austria). The viscosity of the casting solution was measured with a viscometer (Brookfield DV-2 Pro, Middleboro, MA, USA).

### 2.4. Performance Evaluation of Nanofiltration Membranes

The permeation performance of PES/SPES membranes was assessed by measuring pure water flux using deionized water as the feed solution. Rejection performance was evaluated using 1000 ppm NaCl, Na_2_SO_4_ and MgSO_4_ solutions and 10 ppm dye solutions (MEB, MB MO, MY and ST).

For flux and rejection tests, three membrane samples were tested under the same conditions, and the average flux was computed. A membrane with a flux close to the average value was selected for rejection testing. A dead-end filtration system equipped with a nitrogen pressurizer, a digital balance, a sealed water tank and a 300 mL filtration cell (effective diameter: 0.032 m) was used. The filtration procedure was as follows: (1) Compaction: Membranes were pre-compacted at 2 bar for 2 h to stabilize the flux. (2) Measurement of pure water flux: Transmembrane pressure was decreased to 1 bar, and a record was kept of pure water flux every 30 s for at least 40 data points. (3) Rejection test: Deionized water was replaced with salt or dye solutions. The feed solution was stirred at 600 rpm for the minimization of concentration polarization. After stabilization, 15 mL of the filtrate was collected for analysis.

Water permeability (J, L/m^2^ h·bar) was calculated by use of Equation (1):

(1) $$J=\frac{V}{T\times A\times P}$$

where *V* represents permeate volume (L); *T* stands for filtration time (h); *A* denotes effective membrane area (m^2^); and *P* refers to transmembrane pressure (bar).

For salt solutions, the conductivity of the original solution before filtration and the filtrate were measured using a conductivity meter to evaluate the desalination performance of membranes. For dye solutions, an ultraviolet-visible (UV-Vis) spectrophotometer was employed to assess the dye rejection efficiency of membranes.

Rejection rate (*R*) was determined via Equation (2):

(2) $$R=\frac{C_{0}-C_{1}}{C_{0}}\times 100\%$$

where *C*_0_ and *C*_1_ represent the concentrations of feed and permeate solutions, respectively.

The gravimetric method was employed to study porosity and average pore size. Small membrane samples with a diameter of 4.6 cm were cut. Wet membranes were gently wiped to remove excess moisture and then weighed on an analytical balance (W1). Subsequently, the samples were dried at 105 °C for 8 h and weighed again on the analytical balance (W2). This procedure was repeated at least three times for all samples. Average values and standard errors were calculated.

Porosity was calculated using Equation (3):

(3) $$\varepsilon \left(\%\right)=\frac{\left(W_{1}-W_{2}\right)}{A\times L\times \rho }$$

where *A* represents membrane area (m^2^); *L* stands for thickness (m); and *ρ* denotes the density of water (kg/m^3^).

The average pore radius was derived from the Guerout–Elford–Ferry Equation (4):

(4) $$r_{m}=\sqrt{\frac{8\times \mu \times L\times Q\times \left(2.9-1.75\varepsilon \right)}{\varepsilon \times A\times ∆P}}$$

where *μ* represents the dynamic viscosity of water (8.9 × 10⁻^4^ Pa·s); Q stands for water flux (m^3^/s); and ΔP denotes transmembrane pressure (Pa).

## 3. Results and Discussion

### 3.1. Viscosity Measurement of Different PES/SPES Membranes

**Figure 1:**
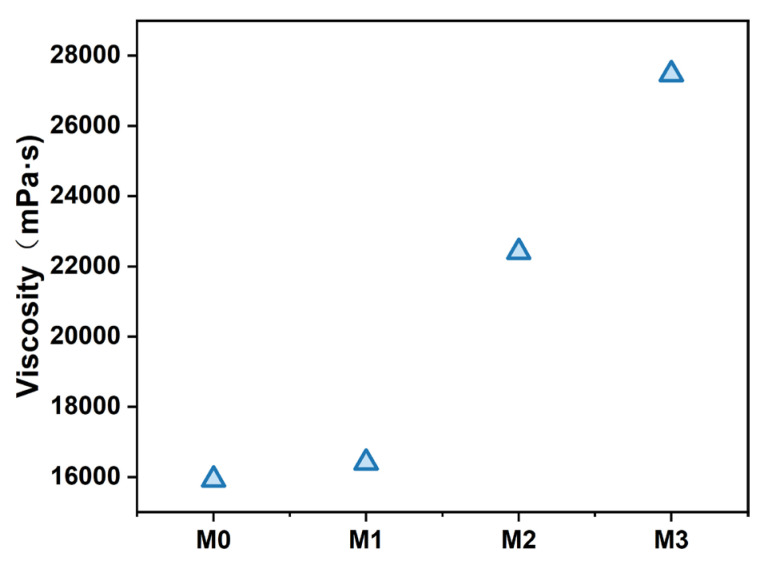
Viscosity of PES/SPES membranes doped with different contents of Ti_3_C_2_T_X_.

The effects of varying Ti_3_C_2_T_X_ contents on the viscosity of the PES/SPES casting solution are illustrated in Figure 1. With the increase of Ti_3_C_2_T_X_ loading, the viscosity of the casting solution exhibited a significant upward trend. This phenomenon indicated that Ti_3_C_2_T_X_ nanosheets were well-dispersed in the DMAc solvent, and abundant hydrophilic functional groups (e.g., hydroxyl (-OH)) on their surface may form hydrogen bonds with PES/SPES, which thereby enhanced intermolecular interactions and increased viscosity. Notably, the change of viscosity directly influenced the solvent–nonsolvent exchange rate during the process of phase inversion.

### 3.2. SEM Characterization of Different Types of PES/SPES Membranes

**Figure 2:**
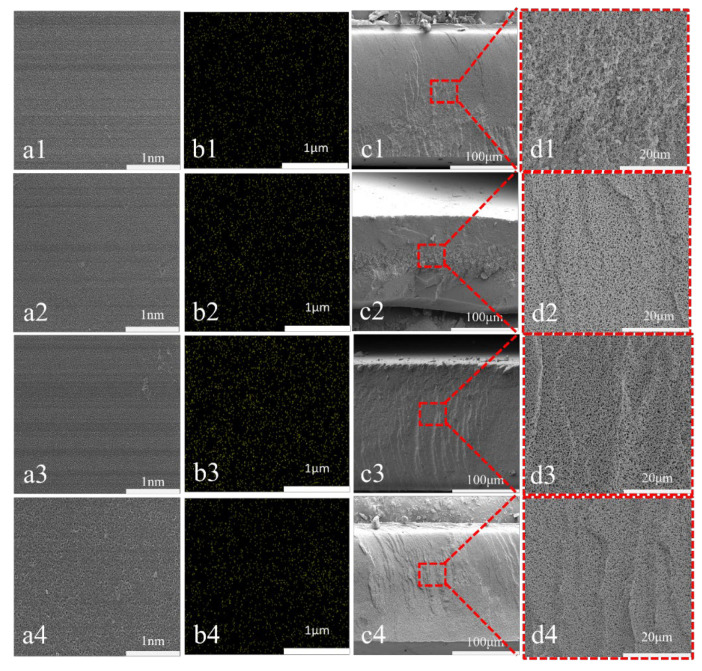
SEM surface images of M0–M3 at 1 nm (**a1**–**a4**); Energy Dispersive Spectroscopy (EDS) diagrams of Ti for M0–M3 at 1 μm (**b1**–**b4**); cross-sectional diagrams of M0–M3 at 100 μm (**c1**–**c4**); cross-sectional diagrams of M0–M3 at 20 μm (**d1**–**d4**).

The surface and cross-sectional SEM images of PES/SPES composite membranes with varying Ti_3_C_2_T_X_ contents (M0–M3), along with the EDS elemental mapping of Ti, are presented in Figure 2. EDS analysis (b1–b4) confirmed the uniform distribution of Ti elements, which indicated the successful incorporation of Ti_3_C_2_T_X_ into the membrane matrix. It was observed from surface images (a1–a4) that the pore density on the membrane surface first increased and then decreased with the increase of Ti_3_C_2_T_X_ content (M0–M3), while pore size initially decreased and subsequently increased. Membranes M1 and M2 exhibited denser and smaller pores, whereas M3 showed larger pores with reduced pore density. This phenomenon was ascribed to the hydrophilic -OH groups on Ti_3_C_2_T_X_ nanosheets, which accelerated the dual diffusion of solvents and nonsolvents during the phase inversion process. Thus, a compact pore structure was formed quickly. However, excessive Ti_3_C_2_T_X_ loading (M3) led to the aggregation of nanosheets, hindered diffusion kinetics and slowing the phase inversion rate, which thereby resulted in sparse macroporous structures [9]. Wei et al. stated that lower polymer concentrations facilitated the formation of finger-like pores near the surface of membranes, while higher viscosity delayed solvent diffusion, shifted the phase inversion process inward, suppressed the formation of finger-like pores and ultimately yielded denser sponge-like structures [10]. This mechanism aligned with the observed pore structure changes in SEM images, where increasing Ti_3_C_2_T_X_ content first reduced and then enlarged surface pore size, with pore density exhibiting the opposite trend.

### 3.3. AFM Characterization of Different PES/SPES Membranes

**Figure 3:**
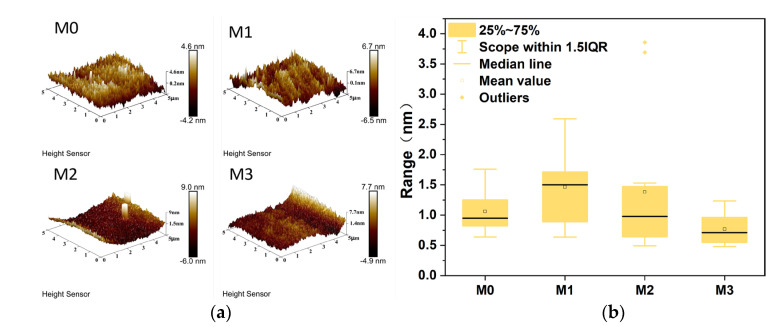
AFM images of M0–M3 (**a**). Roughness of M0–M3 (**b**).

The variations in the surface morphology and roughness of PES/SPES composite membranes with different Ti_3_C_2_T_X_ doping levels via AFM are revealed in Figure 3. It can be found from AFM images (Figure 3a) that the undoped M0 membrane exhibited a relatively smooth surface, but also a trend of initial increase followed by a decrease in surface roughness owing to the introduction of Ti_3_C_2_T_X_ (M1–M3) (Figure 3b). Specifically, M1 showed a notable increase in roughness compared to M0, which was attributable to the hydrogen bonding interactions between the hydrophilic functional groups (e.g., -OH) on Ti_3_C_2_T_X_ nanosheets and the sulfonic acid groups (-SO_3_^−^) in PES/SPES. These interactions promoted localized aggregation, accelerated solvent–nonsolvent exchange during phase inversion and thereby formed microstructural undulations on the membrane surface. Nevertheless, the substantial rise in the viscosity of the casting solution (Figure 1) slowed the phase inversion rate with the further increase of Ti_3_C_2_T_X_ content (M2–M3), which restricted the migration of nanosheets within the polymer matrix and ultimately reduced surface roughness. This phenomenon was aligned with the observed pore structure changes in SEM images (Figure 2(a1–a4)), which indicated that roughness modulation was closely linked to the microstructure of membranes. The results demonstrate that Ti_3_C_2_T_X_ doping regulates surface characteristics through physicochemical mechanisms and provides critical insights for optimizing membrane antifouling and separation performance.

### 3.4. Average Pore Size and Porosity of PES/SPES Membranes with Different Types

**Figure 4:**
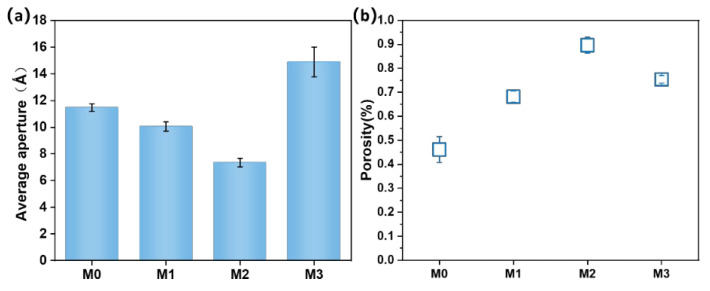
Average pore diameter of M0–M3 (**a**); porosity of M0–M3 (**b**).

The changes in the average pore diameter (a) and porosity (b) of PES/SPES composite membranes with varying Ti_3_C_2_T_X_ doping levels are illustrated in Figure 4. The average pore diameter exhibited a trend of first decreasing and then increasing with the increase of Ti_3_C_2_T_X_ content (from M0 to M3). To be specific, the average pore diameter was 1.15 nm for M0, decreased to 1 nm for M1 (0.1% Ti_3_C_2_T_X_) and then increased to 1.49 nm for M3 (0.5% Ti_3_C_2_T_X_). This phenomenon can be attributed to the dual regulatory effects of Ti_3_C_2_T_X_ on the phase inversion process. At low doping levels (M1–M2), the hydrophilic functional groups (e.g., -OH) on the surface of Ti_3_C_2_T_X_ formed hydrogen bonds with water molecules and accelerated the solvent–nonsolvent exchange rate during phase inversion. This promoted rapid polymer phase separation, which resulted in the formation of a dense and uniform sponge-like structure with reduced pore size and enhanced porosity. However, excessive Ti_3_C_2_T_X_ content (M3) led to a significant increase in the viscosity of the casting solution (Figure 1), hindered solvent diffusion and delayed the phase inversion process, which thus contributed to the formation of larger pores and lower porosity. Furthermore, the synergistic variation in pore size and porosity critically influenced membrane performance. These findings demonstrate that adjusting Ti_3_C_2_T_X_ content can effectively optimize membrane structure and balance permeability and selectivity, which thereby provides theoretical guidance for designing high-performance nanofiltration membranes.

### 3.5. Water Contact Angle and Zeta Potential of Different PES/SPES Membranes

**Figure 5:**
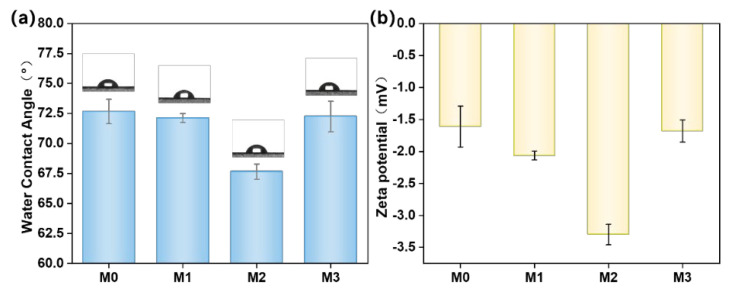
Water contact angle of M0–M3 (**a**); surface zeta potential of M0–M3 (**b**).

The water contact angle (a) and surface zeta potential (b) of PES/SPES composite membranes with varying Ti_3_C_2_T_X_ doping levels are displayed in Figure 5. The water contact angle exhibited a trend of first decreasing and then increasing with the rise of Ti_3_C_2_T_X_ content. Specifically, the undoped M0 membrane displayed the largest contact angle (approximately 72.6°), while the contact angle of the M1 membrane (0.1% Ti_3_C_2_T_X_) significantly decreased to around 40°, which indicated a remarkable enhancement in hydrophilicity. This improvement can be ascribed to the abundant hydrophilic functional groups (e.g., -OH) on the Ti_3_C_2_T_X_ surface, which were partially retained on or within the membrane during phase inversion. Apart from that, the increased surface roughness (Figure 3b) further reduced the contact angle. According to the Wenzel equation, increased roughness amplifies surface hydrophilicity when the contact angle is below 90° [11,12,13]. However, the contact angle slightly rebounded with the further increase of Ti_3_C_2_T_X_ content (M2–M3). This was likely due to the combined effects of reduced porosity (Figure 4b) and decreased roughness (Figure 3b), which diminished the distribution density of hydrophilic groups.

In the tests on surface zeta potential (Figure 5b), all membranes exhibited negative surface charges that primarily originated from the sulfonic acid groups (-SO_3_^−^) of SPES and the negatively charged functional groups (e.g., -OH) on Ti_3_C_2_T_X_ [1,14]. The absolute zeta potential initially increased and then decreased with the rise of Ti_3_C_2_T_X_ content. The M2 membrane demonstrated the highest negative potential (−3.3 mV), whereas the M3 one exhibited a lower value (−1.7 mV). This variation correlated with the reduction in average pore size and the distribution density of Ti_3_C_2_T_X_ on the membrane surface. At lower doping levels, Ti_3_C_2_T_X_ dispersed uniformly and exposed more negatively charged groups, while higher doping levels may induce nanoparticle aggregation, which reduced the effective charged area. Furthermore, the zeta potential trend was consistent with the water contact angle observations, which confirmed the synergistic relationship between surface chemical properties and hydrophilicity. In summary, the incorporation of Ti_3_C_2_T_X_ effectively modulated surface charge and microstructure and significantly optimized the hydrophilicity and separation performance of membranes.

### 3.6. Tensile Properties of Different Types of PES/SPES Membranes

**Figure 6:**
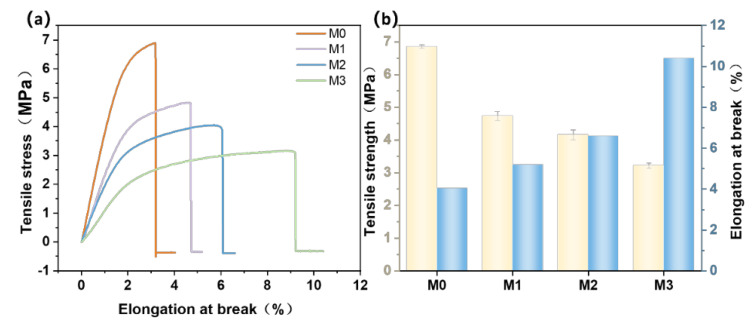
Line diagram of tensile strength and elongation at break of M0–M3 (**a**); histogram of tensile strength and elongation at break of M0–M3 (**b**).

It can be observed from Figure 6 that the tensile strength of PES/SPES composite membranes exhibited a remarkable decrease from 6.86 MPa for M0 to 3.21 MPa for M3 with the increase in Ti_3_C_2_T_X_ doping content (Figure 6b). This phenomenon indicated that the introduction of Ti_3_C_2_T_X_ exerted a negative impact on the mechanical strength of membranes. Previous studies have suggested that insufficient interfacial compatibility between nanosheets and the polymer matrix may reduce stress transfer efficiency despite the excellent flexibility of MXene nanosheets [15], which thereby weakens the overall strength of the blended membranes [1]. Moreover, the dispersion uniformity of Ti_3_C_2_T_X_ might influence the entanglement state of polymer chains, which further exacerbated the decline in mechanical performance.

In terms of elongation at break (Figure 6b), the initial value for M0 was 4% but increased to 10.4% for M3, which indicated a marked enhancement in the plasticity of composite membranes. This change may be put down to the two-dimensional layered structure of Ti_3_C_2_T_X_. The incorporation of MXene might hinder the regular alignment of polymer chains, which increases the sliding space between molecular chains and thereby improves the ductility of membranes. However, the increase in elongation at break was not accompanied by the simultaneous optimization of toughness but led to increased material brittleness. Previous studies have also shown that the addition of soft materials could reduce the mechanical strength of blends [1], and the inherent softness of Ti_3_C_2_T_X_ may contribute to the observed reduction in mechanical strength.

### 3.7. Dye Retention Test of Different PES/SPES Membranes

**Figure 7:**
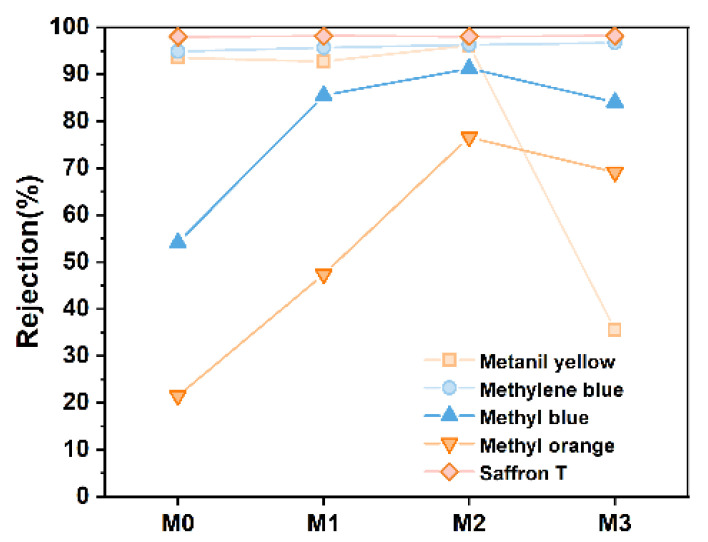
Retention rates of M0–M3 for different dyes.

The retention rates of M0–M3 membranes for five dyes are illustrated in Figure 7. The experimental results demonstrated that the dye retention rates of membranes exhibited an initial increase followed by a decrease with the increase of Ti_3_C_2_T_X_ content. Among these membranes, the M2 membrane (with 0.3 wt% Ti_3_C_2_T_X_) achieved optimal retention performance, which exceeded 90% for MY, MEB and ST. This phenomenon was due to the synergistic effects of pore size sieving and Donnan exclusion. In specific terms, an appropriate amount of Ti_3_C_2_T_X_ enhanced the formation of a dense selective layer during phase inversion and improved pore size sieving efficiency. Concurrently, the increased negative surface charge density (zeta potential, Figure 5b) strengthened electrostatic repulsion (Donnan effect) against positively charged dyes like MEB. Of note, their retention rates differed significantly, notwithstanding similar molecular dimensions (approximately 4.8 × 14.9 Å for MO and 5.8 × 14.4 Å for MB, Table 2). This discrepancy arose from charge interactions; MO (positively charged) experienced electrostatic attraction with the negatively charged membrane surface, which reduced retention efficiency, while MB (negatively charged) benefited from enhanced electrostatic repulsion. Furthermore, the decreased retention rate of the M3 membrane (0.5 wt% Ti_3_C_2_T_X_) may result from increased pore size, broader pore size distribution and reduced surface charge density, which collectively weakened sieving and electrostatic effects.

To sum up, Figure 7 highlights the dual regulatory role of Ti_3_C_2_T_X_ content in optimizing membrane retention performance and emphasizes the importance of synergistic mechanisms between pore size sieving and Donnan exclusion in dye separation [12,18,19,20,21,22]. These findings provide critical insights for the design of high-performance composite membranes.

### 3.8. Measurement of Water Flux and Salt Ion Retention of Different PES/SPES Membranes

**Figure 8:**
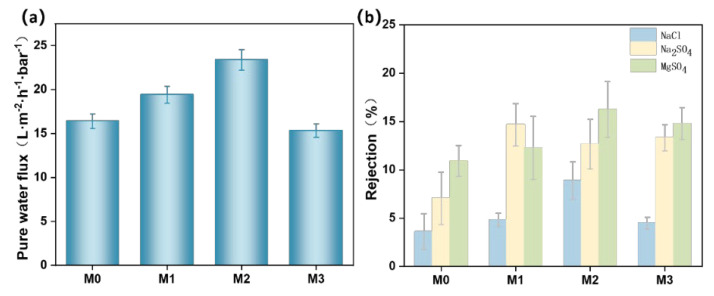
Pure water flux of M0 to M3 (**a**); salt ion rejection rates of M0 to M3 (**b**).

The variations in the pure water flux (a) and salt ion rejection rates (b) of PES/SPES composite membranes with different Ti_3_C_2_T_X_ doping levels are illustrated in Figure 8. Pure water flux initially increased and then decreased with the addition of Ti_3_C_2_T_X_ (Figure 8a). The initial enhancement in flux was attributed to the hydrophilic groups (e.g., -OH) on Ti_3_C_2_T_X_. This accelerated the dual diffusion of solvents and water during phase inversion, which led to the formation of a sponge-like structure with higher pore density (Figure 2). However, excessive Ti_3_C_2_T_X_ content (e.g., M3) significantly increased the viscosity of the casting solution (Figure 1) and inhibited the solvent exchange rate during phase inversion. This resulted in reduced pore density and enlarged pore size (Figure 4a), which ultimately lowered pure water flux.

Salt rejection rates also exhibited a trend of first increasing and then decreasing (Figure 8b) in the following order: MgSO_4_ > Na_2_SO_4_ > NaCl. This behavior can be explained by two mechanisms: size sieving and the Donnan effect. First, Mg^2^⁺ has a larger hydrated radius than Na⁺, which makes MgSO_4_ more easily rejected by the dense layer of the membrane surface. Second, the negatively charged membrane surface (due to the sulfonic acid groups in SPES and the -OH groups in Ti_3_C_2_T_X_, Figure 5b) exerted stronger electrostatic repulsion on multivalent anions (e.g., SO₄^2^⁻), which thereby improved the rejection of MgSO_4_ and Na_2_SO_4_. This is in line with previous studies [4]. Nevertheless, the enlarged pore size (Figure 4a) and reduced surface charge density (Figure 5b) weakened both sieving and electrostatic effects with the further increase of Ti_3_C_2_T_X_ content (M2–M3), which led to a decline in salt rejection.

### 3.9. Flux Recovery Test of Different Types of PES/SPES Membranes

**Figure 9:**
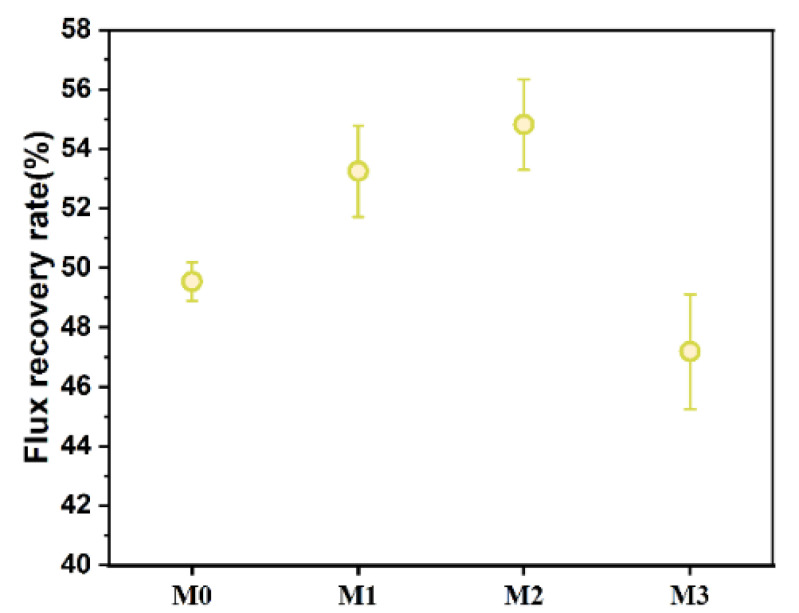
Flux recovery rates of M0–M3.

It can be seen from Figure 9 that the flux recovery rates of composite membranes exhibited an initial increase and then a decrease with the addition of Ti_3_C_2_T_X_. From M0 to M2, the flux recovery rate improved from 49.5% to 54.8%. This enhancement was primarily attributed to the abundant hydrophilic groups (e.g., -OH) on the surface of Ti_3_C_2_T_X_, which increased membrane hydrophilicity, promoted the wetting effect of water molecules on the membrane surface, reduced the adsorption of contaminants (e.g., proteins and organic molecules) and thereby improved antifouling performance [23,24]. Beyond that, the negative surface zeta potential (Figure 5b) under moderate Ti_3_C_2_T_X_ content further enhanced the electrostatic repulsion effect and effectively inhibited the attachment of negatively charged pollutants. However, the flux recovery rate decreased from 54.8% to 47.2% from M2 to M3. This may be because excessive Ti_3_C_2_T_X_ content reduced the density of surface -OH groups and weakened zeta potential, which diminished both hydrophilicity and electrostatic repulsion. Concurrently, increased pore size (Figure 4a) and altered porosity (Figure 4b) may allow pollutants to penetrate deeper into membrane pores, which exacerbated irreversible fouling. This phenomenon was in alignment with the reported positive correlation between membrane hydrophilicity and antifouling performance [23,24], which indicated that the doping of Ti_3_C_2_T_X_ must be optimized within a reasonable range to balance flux recovery and membrane stability.

### 3.10. Antibacterial Test of Different Types of PES/SPES Membranes

**Figure 10:**
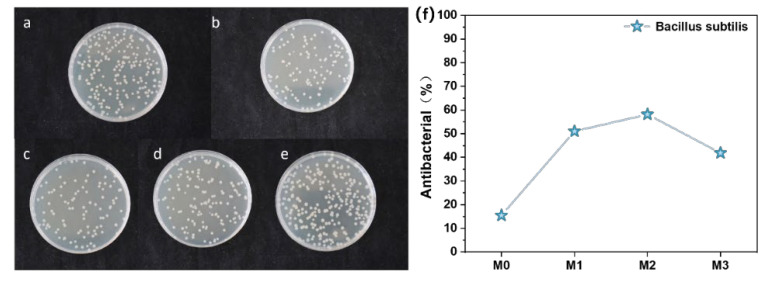
Bacterial inhibition of M0–M3 against *Bacillus subtilis* (**a**–**e**); control group without membrane (**e**); bacteriostatic rate of M0–M3 against *Bacillus subtilis* (**f**).

**Figure 11:**
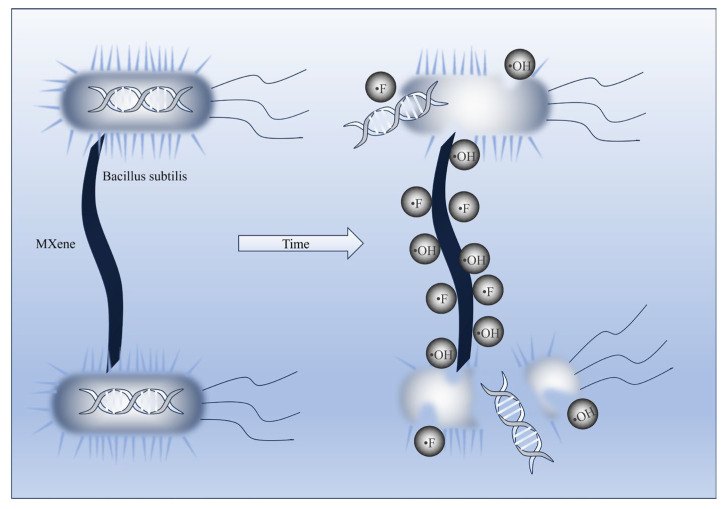
Mechanistic diagram of MXene antibacterial activity.

According to the bacteriostatic effects and rates of M0–M3 against *Bacillus subtilis* shown in Figure 10a–f, the antibacterial performance of composite membranes significantly improved with increasing Ti_3_C_2_T_X_ content. The undoped M0 membrane exhibited a bacteriostatic rate of only 15%, while the M2 one doped with 0.3% Ti_3_C_2_T_X_ achieved a bacteriostatic rate of 58%, which indicated that the introduction of MXene effectively enhanced the antimicrobial activity of membranes. This improvement can be attributed to the unique physicochemical synergistic mechanisms of MXene. Firstly, the hydrophilic functional groups (e.g., -OH) on MXene nanosheets enhanced adhesion to bacterial cell membranes via hydrogen bonding and formed a physical trapping effect that restricts the nutrient exchange required for bacterial proliferation [25,26,27,28,29,30,31]. As posited by physical trapping theory, the hydrophilic groups and anions on the MXene surface strengthened interactions with bacterial cell membranes. MXene can adhere to bacteria and trap or encapsulate them into aggregates. This thus limited intracellular nutrient exchange and proliferation, which led to reduced bacterial viability or death due to nutrient deprivation [25]. More than that, the enhanced interaction between the -OH groups on the surface of composite membranes and water molecules formed a hydration layer, which reduced direct bacterial contact and caused bacterial inactivation [26,32]. Secondly, the sharp edges of MXene nanosheets can directly damage bacterial cell membranes through physical cutting, which results in the leakage of intracellular contents (as illustrated in Figure 11) [33,34,35]. Furthermore, titanium dioxide (TiO_2_) nanocrystals generated during MXene oxidation can induce reactive oxygen species (ROS) production and accelerate bacterial death via oxidative stress responses [36,37,38]. Oxidative stress primarily arose from the hydrogen bonds formed between oxygen-containing groups on MXene nanosheets and cell membranes. These bonds blocked nutrient uptake and induced ROS generation, which led to bacterial inactivation and growth inhibition [33,39]. Additionally, the reactive functional groups on the membrane surface (e.g., Ti-F in alkaline environments) may form conductive bridges across lipid bilayers, which transfer reactive electrons to the external environment and trigger apoptosis [34]. It is worth noting that the bacteriostatic rate of the M3 membrane slightly decreased compared to that of the M2 membrane. The possible reason is that excessive Ti_3_C_2_T_X_ caused agglomeration, which reduced the effective contact area and dispersibility of MXene nanosheets [7,38]. This observation aligned with the reduced hydrophilicity of the M3 membrane (Figure 5a), which further confirmed the critical role of MXene surface group distribution in antibacterial performance.

### 3.11. Performance Comparison Between M2 Membrane and the Membrane Reported in the Literature

Table 3 presents a comparative analysis between the established commercial or synthetic membranes and the M2 membrane regarding their efficacy in dye removal. From the data, it is evident that the overall performance of the M2 membrane surpasses that of the established commercial membranes.

## 4. Conclusions

In the present study, a novel PES/SPES nanofiltration membrane was prepared by incorporating Ti_3_C_2_T_X_ nanosheets into the casting solution via the NIPS method. The effects of Ti_3_C_2_T_X_ content on membrane morphology and performance were investigated systematically. The results are summarized as follows:

(1) Physicochemical properties: The incorporation of Ti_3_C_2_T_X_ significantly influenced the physicochemical properties of the membrane. With the increase of Ti_3_C_2_T_X_ content, the water contact angle of the nanofiltration membrane first decreased and then increased. In addition, the changes in phase inversion rate induced by Ti_3_C_2_T_X_ content led to corresponding variations in the average pore size and porosity of the membrane.
(2) Separation performance: The addition of Ti_3_C_2_T_X_ notably affected membrane performance. As Ti_3_C_2_T_X_ content increased, key performance metrics such as water flux, dye rejection rate, flux recovery rate and salt ion rejection rate initially improved and then declined. These findings provide valuable insights for the optimization of membrane technology in salt/dye separation applications.
(3) Antibacterial performance: Ti_3_C_2_T_X_ doped membranes exhibited significant antibacterial advantages. The bacteriostatic rate against *Bacillus subtilis* increased from 15% (the M0 membrane) to 58% (the M2 membrane), which demonstrated enhanced antimicrobial efficacy.
(4) Advantages and disadvantages: The incorporation of a small amount of Ti_3_C_2_T_X_ into the hybrid membrane offers several advantages, including an increased flux alongside an enhanced rejection rate, thereby overcoming the trade-off effect. Additionally, the antibacterial performance of the hybrid membrane is significantly improved. However, a disadvantage arises as the amount of Ti_3_C_2_T_X_ increases, leading to a continuous decline in the mechanical properties of the membrane, which may affect its service life. Despite the limited usage of Ti_3_C_2_T_X_, the improvement remains evident. Moreover, the one-step phase inversion method enables preparation of the membrane, indicating potential for industrialization.

## Figures and Tables

**Table 1:** Compositions of the casting solution used for membrane preparation.

Membrane	Casting Solution Composition (wt%)				Ti_3_C_2_T_X_ Mass Ratio (%)
	PES	SPES	DMAc	Ti_3_C_2_T_X_	
M0	5.4 g	3.6 g	21 g	0 g	0%
M1	5.4 g	3.6 g	21 g	0.03 g	0.1%
M2	5.4 g	3.6 g	21 g	0.09 g	0.3%
M3	5.4 g	3.6 g	21 g	0.15 g	0.5%

**Table 2:** Properties of dyes.

Dye Molecule	Electrical Property	Molecular Weight g/mol	Maximum Absorption Wavelength (nm)	Molecular Size (Ǻ)
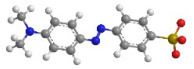 MO	Positive	327.33	463	4.8 × 14.9 (Ǻ) [16]
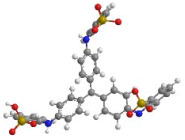 MB	Negative	799.8	664	5.8 × 14.4 (Ǻ) [16]
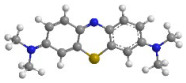 MEB	Positive	373.9	664	15.805 × 7.91 × 4.019 (Ǻ) [17]

**Table 3:** Performance comparison between M2 membrane and the membrane reported in the literature [40].

Membrane	Permeability (LMH bar^−1^)	Types of Dye	Dye Rejection
M2	23.4	Safranine T Methylene blue	98 96.3
TFN-mZIF2 [41]	14.9	Reactive blue 2 Reactive black 5	99.2 99.0
PVA/PSSNa [42]	8.3	Congo Red	99.7
Sepro NF 6 [43]	10.5	Congo red Direct red 80 Direct red 23	99.9 99.9 99.9
GO/FLG [44]	6.7	Rhodamine B Acid Blue 9	8.0 96.0
NF90 [45]	20.2	Congo red	99.6
SMA-PEI/PES [46]	23.0	Congo red Rhodamine B Methylene blue	99.4 44.7 7.3
SC-MXene [45]	9.0	-	-
TFC-5 [40]	20.9	Congo red Reactive blue 19 Methyl blue	99.429% 99.02% 98.84%

## Data Availability

The original contributions presented in this study are included in the article. Further inquiries can be directed to the corresponding author.
